# A Controlled Trial Evaluating the Impact of a Home-Visiting Program on Maternal Disruptive Communication in a Vulnerable Population

**DOI:** 10.3390/children9081166

**Published:** 2022-08-03

**Authors:** Susana Tereno, Tim Greacen, Antoine C. Guedeney

**Affiliations:** 1 Département de Psychologie, Université Rouen Normandie, CRFDP, EA 7475, CEDEX, 76821 Mont Saint Aignan, France; 2 Laboratoire de Recherche en Santé Mentale et Sciences Humaines et Sociales, GHU Psychiatrie et Neurosciences, 75014 Paris, France; timgreacen@gmail.com; 3 Institut National de la Santé et de la Recherche Médicale, U669 PSIGIAM, Service de Pédopsychiatrie, Hôpital Bichat Claude-Bernard, 75018 Paris, France; guedeney@free.fr

**Keywords:** controlled trial, vulnerability, maternal disruptive communication, attachment, home-visiting, early intervention

## Abstract

Attachment disorganization is a significant high-risk factor for infant mental health. Its association with high-risk psychosocial contexts has been clearly identified, but the link between these difficult social contexts and maternal disruptive communication has been poorly explored. The CAPEDP (Compétences Parentales et Attachement dans la Petite Enfance; Parental competences and attachment in early childhood) study assessed the effects of a manualized home-intervention on the mental health of children and its major determinants. In this controlled trial, 440 young, first-time mothers belonging to socially vulnerable populations were recruited. Mothers in the intervention group received psychological support from the 27th week of pregnancy through to their child’s second birthday, while both groups received assessment visits at the 3rd, 6th, 9th, 18th, and 24th months of age of the child and benefited from assistance by the research team. When the children reached 12 months of age, an ancillary study, the CAPEDP-Attachment (*n* = 119) evaluated the effects of this intervention on attachment. The current paper describes the program’s impact on this subsample concerning maternal disruptive behavior, while exploring the role of socioeconomic risk factors. Our results showed that: (a) mothers in the intervention (IG) group presented significantly less disruptive communication than those in the control group (CG), even though the CG received a significant level of care over and above that which is available to the public in the French health system as ‘care as usual’; (b) having a “low income” and “having given birth prematurely” was associated with maternal disruptive communication; and (c) the intervention impact increased when the model was adjusted for these two variables. Results suggest that attachment focused intervention programs should invest both maternal interactional skills and social and economic vulnerability.

## 1. Introduction

The scientific literature suggests that attachment style is an important determinant of infant mental health, since it influences the child’s ability to manage stressful situations, a fundamental component of social and emotional development in the early years [1]. Caregiver sensitivity is indicative of attachment security, but disorganization seems mostly to be predicted by parental disruptive interactions. Although disruptive parental behaviors are not limited to multiple-risk family environments, the accumulation of risk factors reduces parental psychic availability and stress tolerance, consequently exacerbating the loss of parental self-control [2]. Independently of the type of risk factors involved, researchers have found that cumulative risk is associated not only with an increase in negative outcomes [3,4,5,6], but also with a greater prevalence of clinical problems [6,7]. Cyr et al. [8] describe multiple possible pathways to attachment disorganization, including both parental abusive maltreatment or neglect of the child. Researchers have sought to address this question by implementing intervention programs that target multi-risk families, as these are the ones known to be the most prone to attachment disorganization and have demonstrated their preventive efficacy. The present paper describes the CAPEDP program’s impact on the CAPEDP-Attachment subsample regarding maternal disruptive behavior and taking into account the role of different socioeconomic risk factors.

### 1.1. Maternal Disruptive Communication in Vulnerable Populations

Lyons-Ruth et al. [7] define *maternal disruptive communication* as all maternal interactive behavior that does not effectively respond to an infant’s attachment needs, one that does not consequently deactivate his/her attachment system, contributing, as a result, to the development of a disorganized attachment pattern. Initially, researchers, such as Main and Hesse [9] and Solomon and George [10], categorized disruptive communication into two different types: *frightening and frightened* parental behavior; subsequently, Lyons-Ruth et al. [7] divided these two general categories into five dimensions, assessed by their AMBIANCE coding system: *(1)* *negative-intrusive behavior* describes behavior that is frightening or threatening, that communicates a hostile attitude towards the infant, or interferes with the infant’s movements, such as rough handling of the infant, negative imputations against the child, mocking, and teasing; *(2)* *role confusion* designates behavior that prioritizes the parent’s needs over the infant’s, such as asking for reassurance or affection from the infant when the latter is in a state of distress, or, more rarely, sexualized behavior towards the infant; *(3)* *disorientation* is parental behavior that appears frightened, dissociated, or affectively odd, such as unusual changes in pitch and intonation of voice, unchanging ”flat” affect, and stiff or awkward body postures when interacting with the infant; *(4) affective communication errors* include contradictory communication or failure to respond to an infant’s cues, especially those related to comfort, for example, verbally inviting the infant to approach followed by physical distancing, or leaving the infant crying on the floor without response; and *(5) withdrawal* is behavior that communicates reluctance to interact fully with the infant, such as walking around the infant when reuniting with it, hesitating before responding to an infant’s cue, quick pick-up and put-down when the infant is distressed, or interacting silently with the child.

Although the relation between child maltreatment and disorganization has been the object of numerous studies [2,11,12,13], strangely, the association between socioeconomic factors and disorganization has received less attention. Socioeconomic risks are pervasive, often prolonged, with a propensity to co-occur and to accumulate in the same families and individuals, creating a precarious context that increases the child’s distress and insecure/disorganized attachment [6,14,15]. In a high-risk sample, Shaw and Vondra [16] found that, in families with at least three or more stressors, the greater the number of risk factors, the more likely the children were to develop insecure attachment. With low-risk subjects, research conducted by Belsky [17] and Belsky et al. [14] also supports the cumulative risk hypothesis. In their meta-analyses, Cyr et al. [7] showed that the presence of risk is sufficient to increase attachment insecurity and disorganization, but that cumulative risk factors seem to specifically intensify disorganized attachment.

### 1.2. Intervention Programs Targeting Maternal Disruptive Communication

Intervention programs targeting multi-risk families, those most prone to attachment disorganization, have shown their preventive efficacy. Of all home-based interventions, those conducted jointly by nurses and infant mental-health workers have been found to yield the best results, particularly in reducing externalizing behavior problems [18]. In 2003, Bakermans-Kranenburg et al. [19] performed a meta-analysis on the impact of attachment-focused interventions, and came to the conclusion that, in general, these interventions increased attachment security by enhancing maternal sensitivity, with shorter interventions with more precise goals seeming to be more effective than lengthy, multi-focal programs. Moss et al. [20] highlight the need for extensive training and supervision for intervention professionals. In their study, as in the study presented in the present paper, trained and supervised psychologists were the principal actors. 

Despite the importance of these studies, their correlational nature does not allow us to understand the processes that explain the positive effects of these intervention programs. Moran et al. [21] underline the importance of differentiating the role of maternal sensitivity from that of maternal disruptive communication, the first being typically associated with infant security and the second rather with disorganization. Furthermore, despite the fact that the results show an association between socioeconomic vulnerability and infant attachment disorganization, no research to this date has set out to explore the impact of socioeconomic risk on maternal disruptive behavior.

In France, the CAPEDP study, a randomized controlled trial on promoting infant mental health, abiding by the CONSORT guidelines was launched in Paris in 2006. This project intended to address infant mental-health problems by reducing maternal postpartum depression and promoting parenting skill and social and professional integration [22]. The CAPEDP-Attachment (CAPEDP-A) is an ancillary study of the CAPEDP project that evaluated a subsample of the participant families with regard to child attachment security and disorganization, as well as maternal disruptive communication and mentalizing skills [23]. Results on the infants’ attachment quality have been published elsewhere [23,24]. The current paper specifically aims to analyze the CAPEDP program’s impact on the CAPEDP-A subsample with regard to reducing maternal disruptive communication whilst taking into account the role of socioeconomic risk factors.

## 2. Materials and Methods

### 2.1. Participants 

Participants in the CAPEDP general study (*n* = 440) were recruited at maternity wards in 10 public hospitals in the Paris area during prenatal care check-ups. Inclusion criteria included being a young (<26), first-time mother, less than 27 weeks pregnant, with one or more of the following psychosocial vulnerability factors: (1) having less than 12 years of education, (2) declaring the intention to raise the child without the father, and (3) having a low income. Women who had already had a social or medical follow-up for reasons other than those described above were not included, in order to avoid bias on the effect of the intervention. After inclusion, the women were then randomly assigned to either the “care as usual group” or to the “intervention group”, the latter benefiting from a reinforced CAPEDP home-visiting program [24]. The control group received “care as usual” which, in France, involves community-based mother–child support and prevention services (Protection Maternelle et Infantile; PMI). Families can use PMI services for free up until their child’s third birthday, a service that includes mental health support, if desired and requested, or if recommended by a PMI nurse. However, PMI nurses are not trained in screening or treating mental health issues and related stressors and 60% of the high-risk families receive home visitations limited to 1 per family. In the present study, the intervention group benefited from both this “care as usual” and the CAPEDP home-intervention program.

Families participating in the main CAPEDP trial were systematically invited to participate in the CAPEDP-A sub-study when their child reached 12 months of age. Mothers who agreed to participate with their child signed an informed consent form and made an appointment for a two-hour assessment session in the CAPEDP Attachment Lab. A total of 119 mothers agreed to participate. Each received a EUR 50 voucher to cover their eventual expenses. The sociodemographic characteristics of women who were still in the study when their child reached 12 months of age but who declined to participate in the CAPEDP-A sub-study (*n* = 248) were not significantly different from those of the women who accepted (*n* = 119) (see Table 1). CAPEDP-A received ethical approval from the *Comité de Protection des Personnes Ile de France IV*, the Institutional Review Board, as well as from the *Commission Nationale de l’Informatique et des Libertés* (CNIL, 907255), with Clinical Trial Registration Number NCT00392847. 

### 2.2. Procedures

#### 2.2.1. Assessment

At baseline and at follow-up visits, four levels of psychosocial vulnerability were assessed for all participants: (1) socioeconomic vulnerability; (2) psychological vulnerability; (3) parenting vulnerability; and (4) infant vulnerability. 

As mentioned above, when the infants were between 12 and 17 months of age, all mothers in the CAPEDP-A study participated in a two-hour assessment procedure evaluating *infant attachment quality*, *maternal disrupting communication,* and *maternal auto-reflexive function*. In the CAPEDP-A study, a specific attachment assessment team, blind to the CAPEDP general intervention, received training on the procedures and instruments used to evaluate attachment issues in mother–infant dyads. Parallel coding teams for each instrument were established to ensure a blinded coding system, where coders were aware neither of the subject’s group nor of other assessment results. 

#### 2.2.2. The Intervention

CAPEDP intervention psychologists visited (for a duration of approximately 60 min) the families remaining in the study until their child reached 24 months of age, on average 6 times during the prenatal period (starting from the 27th week of pregnancy), 8 times during the first 3 months of the child’s life, 15 times when the child was between 4 and 12 months of age, and another 15 times during the child’s 2nd year. For additional details concerning the manualized CAPEDP intervention may be found elsewhere [25].

The intervention team was composed of female psychologists similar in age to that of the mothers included in the study, with specific training in working alliance skills, early child development, and health promotion during pregnancy. The assessment team was composed of four psychologists. The intervention team members were blinded to assessment results. This was possible because they were not the same psychologists of the assessment team and, therefore, had no access to the dyads assessment results. Assessment visits at the 27th week of pregnancy and when the child was 3, 6, 12, 18, and 24 months old were performed for all 440 participants. The mean number of total home visits performed by intervention psychologists for each family was 44. Telephone calls between visits could be made as often as necessary. The intervention was manualized but adjusted to each family’s needs (for a more detailed description of the study protocol, see [25]).

Regarding attachment issues, the CAPEDP-A research team developed specific tools to support the intervention team [23]. The overall aim of the attachment-focused intervention was to increase maternal sensitivity (to detect and provide adequate/rapid responses to the child’s needs) and mentalizing skills (to identify their own and their infant’s intentions and emotional states). Intervention also sought to decrease disrupted maternal communication (to detect, prevent, and repair disrupted maternal affective communication), as well as disorganized behavior (to detect and emotionally repair disorganized behavior). To address these aims, all home-visiting psychologists received specific training before the intervention began. 

They were also provided with two manuals, intended to structure their interventions: the first focusing on attachment and the second on oppositional behavior. In order to underline the importance of early attachment relationships, a pamphlet centered on infant emotional development was presented to and discussed with the families by the intervention psychologists. Specific training was provided to the psychologists on the use of video to help parents reflect on their parenting experiences and practices, not only in order to promote attachment security in infants from 6 to 9 months of age but also to teach parents how to handle oppositional behavior in children aged between 12 and 15 months. To ensure treatment fidelity, all home-visiting psychologists received weekly individual supervision with senior clinicians, group supervision by the CAPEDP principal investigator (AG) twice a month, and monthly video feedback supervision by the CAPEDP-A project manager (ST). When CAPEDP-A evaluations took place, at around the children’s first birthday, families in the intervention group had already received a mean of 29 home visits (for a detailed description of the attachment intervention, see [23,25]).

### 2.3. Measures

The primary outcome was measuring disruptive caregiver behavior, using the *Atypical Maternal Behavior Instrument for Assessment and Classification* (AMBIANCE; [26]), a tool that codes disruptive caregiver behavior during videotaped caregiver–infant interactions, in this case using the *Strange Situation Procedure* videos (SSP; [27]), a laboratory paradigm involving a series of eight 3 min, increasingly stressful episodes for 12–18 month-olds. After a recording, all instances of disruptive communication are identified and a frequency score is derived for each of the five dimensions of the AMBIANCE Scale: affective communication errors, role/boundary confusion, fearful/disorientation, intrusive/negative, and withdrawing behavior. The AMBIANCE coding system also involves a 7-point scale assessing the global level of disruptive communication, with a score of 1 corresponding to high normal behavior, 3 to low normal behavior, 5 to clear evidence of disruption in affective communication, and 7 to disruptive communication with few or no ameliorating behaviors. The global level of disruptive communication is based on the frequency and intensity of such communication as displayed by the caregiver. A binary classification is then attributed, where scores of 5 or above are classified as “disruptive” and scores less than 5 as ”non-disruptive” [24]. All four coders in the assessment team were trained by Elisabeth Bronfman and Karlen Lyons-Ruth, all obtaining a reliability level superior to 80% for the binary classification. Overall, 50% of the videos were coded with AMBIANCE by a second independent rater, and inter-coder reliability for the two-way classification was 83%. 

In order to identify risk factors levels at inclusion (baseline), variables were grouped according to four types of risk involved:

(1) *Socioeconomic risk,* composed of four sub-categories: educational level lower than 9 years of schooling; low income (defined as being sufficiently poor to be eligible for free health care); first generation immigrant; and single parenthood (defined as intending to bring up the child without the presence of the child’s father).
(2) *Psychological risk,* composed of three sub-categories: Edinburgh Postnatal Depression Scale > 11 (EPDS; [28]; Symptom Check List-90 global score > 0.80 (SCL-90; [29]); and tobacco and/or alcohol use during pregnancy.
(3) *Parenting risk,* composed of four sub-categories: perinatal complications; unplanned/unwanted pregnancy; early loss of attachment figure (when the woman was less than 11 years old); and being under 20 years of age.
(4) *Infant risk,* composed of two sub-categories: premature birth and tobacco and/or alcohol use during pregnancy.

Subjects were coded for each variable if they met at least one of the sub-categories in question.

### 2.4. Statistical Analysis Procedures

Maternal disruptive behavior and risk-factor variables were compared using Pearson’s chi-square test. When required, the comparison of independent variables used a t-test. Whenever risk-factors variables were significantly different between groups analysis were adjusted to avoid potential imbalances in results. The impact of the program on maternal disruptive communication was assessed using logistic regression analysis. Effect-sizes were calculated using inverted Odds Ratio (OR) values following Andrade’s (2020) recommendations that standardized mean differences (SMD) should be only used in meta-analytic designs, advising the use of isolated OR for studies such as the present one. Analyses were considered significant at the 5% confidence limit using two-sided tests. Missing data concerning the endpoints were handled by conducting multiple imputation and sensitivity analyses. Statistical Analysis Software (SAS) version 9.2 (SAS Institute Inc., Cary, NC, USA) was used for all statistical analyses. 

## 3. Results

### 3.1. CAPEDP-A Flowchart

**Figure 1:**
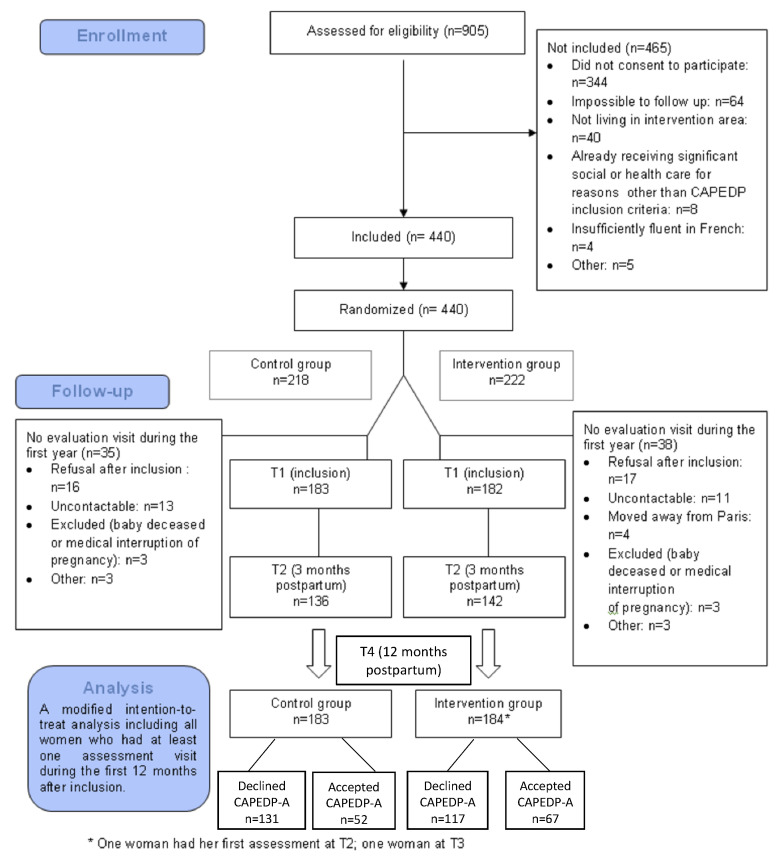
CAPEDP-A flowchart diagram.

Figure 1 presents the Flowchart of the CAPEDP-A study. As can be seen, of the 440 subjects included in CAPEDP, the modified intention-to-treat analysis population (subjects who had at least one assessment visit during the first year following delivery) was 367 (83.4%), including 365 who had a visit at T1 (inclusion); two mothers received their first visit at T2 (3 months postpartum) and T3 (6 months postpartum), respectively [30]. From the 367 women invited to participate in the CAPEDP-A sub-study when their child was 12 months old (T4), 248 declined to participate and 119 accepted. 

### 3.2. Risk Factors

Of the 119 mother–infant dyads recruited into the CAPEDP-A protocol, 114 (96%) had their Strange Situation videos coded using the AMBIANCE system. Five were not coded due to technical problems with the video recorders. A total of 63 of these dyads were in the intervention group and 51 in the control group (usual care). Socio-demographic variables were categorized into four types: *socioeconomic*, *maternal psychopathology*, *parenting,* and *infant risks* (Table 2):

(1) The average age of the infants when assessment took place was 14.2 (SD = 2.8) months;
(2) The mothers’ average age at inclusion was 22.3 (SD = 2.5; median age = 23.0 (20.0–24.5));
(3) Almost half of the mothers (*n* = 57; 47.9%) were first generation immigrants;
(4) 15.1% (*n* = 18) had less than 9 years of education, but a large majority (*n* = 100; 83%) had less than 12 years of education;
(5) In total, 39.5% (*n* = 45) of households had a monthly income of less than EUR 840;
(6) Overall, 40.3% (*n* = 48) declared that they were not living in a couple, and 59.7% (*n* = 71) were married to or living with the child’s father;
(7) Overall, 24.2% (*n* = 29) of mothers declared themselves to be single parents (intending to raise their child without the father);
(8) Overall, 42.9% (*n* = 51) were sufficiently poor to be eligible for free, state-funded healthcare.

Few differences were observed between the intervention and control groups on socio-demographic variables at inclusion. The only two variables that proved to be significantly different were the following:

(1) The number of women under the age of 20 was lower in the intervention group (*n* = 20; 37.7%) than in control group (*n* = 14; 20.9%); χ^2^ (1) = 6.52; *p* = 0.01);
(2) Women in the intervention group declared more tobacco and/or alcohol use during pregnancy (*n* = 24; 35.8%) than control-group mothers (n = 9; 17.3%; χ^2^ (1) = 5.01; *p* = 0.03).

To avoid any bias induced by these imbalances between the groups, all the subsequent analyses were adjusted for these two factors.

With regard to the risk-factor accumulation, 75.6% (*n* = 90) of women presented at least three risk factors and 37.8% (*n* = 45) at least five, with no differences between groups. 

### 3.3. Maternal Disruptive Communication

#### 3.3.1. Maternal Disruptive Communication and Risk Factors

In order to identify factors in the mothers’ sociodemographic situations associated with maternal disruptive communication (MDC), a logistic regression was performed (adjusted for the group to which the mothers belonged; see Table 3). 

Univariate analyses showed that mothers with MDC (assessed at infants’ 12 months) were more likely at inclusion to have:

(1) Benefited from free health care (χ^2^ (1) = 4.21; *p* = 0.04; disruptive mothers: *n* (%) = 26 (54.2%) vs. non-disruptive mothers: *n* (%) = 22 (33.8%);
(2) Given birth prematurely (χ^2^ (1) = 4.24; *p* = 0.04; disruptive mothers: *n* (%) = 6 (13.0) vs. non-disruptive mothers: *n* (%) = 2 (3.2)).

When analyzing risk factors by their number independently of their type, the present analysis revealed that mothers with disruptive communication were more likely to present (at inclusion):

(1) A higher number of risk factors (F(1) = 6.74; *p* = 0.01; disruptive mothers: mean (SD) = 4.4 (2.1) vs. non-disruptive mothers: mean (SD) = 3.4 (2.0));
(2) At least five risk factors (χ^2^ (1) = 5.61; *p* = 0.02; disruptive mothers: *n* (%) = 24 (50.0) vs. non-disruptive mothers: *n* (%) = 20 (30.3)).

No significant difference in the number of intervention visits was observed (disruptive mothers: *n* (%) = 18.5 (7.05) vs. non-disruptive mothers: *n* (%) = 19 (7.7)).

#### 3.3.2. Impact of Intervention on Maternal Disruptive Communication

As mentioned above, to avoid any bias induced by imbalances between the groups, all the subsequent analyses were adjusted for mothers’ age (less than 20 y-o) and tobacco/drug use during pregnancy, the only two risk factors significantly different between groups.

A chi-square test was performed to assess differences in maternal disruptive communication (MDC) between the groups. Mothers in the intervention group had significantly lower levels of MDC than the control-group mothers (χ^2^ (1) = 4.45; *p* = 0.04). One in three (33.3%; *n* = 20) of the intervention-group mothers presented MDC, compared to more than one in two (52.9%; *n* = 27) in the usual-care group (Table 4). 

A logistic regression analysis showed that mothers in the CAPEDP home-visiting program were 56% less likely to present MDC than mothers in the control group (OR = 0.44; effect-size = 2.27; *p* = 0.04). 

In order to propose an explicative model to understand the impact of the CAPDEP intervention on MDC, a logistic regression analysis was conducted, taking into account the two risk factors identified as being significantly associated with this behavior at inclusion (*low income* and *giving birth prematurely*). Table 5 shows that when these two variables are taken into account in the regression analysis, MDC is 69% lower in the intervention group (OR = 0.31; effect-size = 3.23; *p* = 0.04) compared with the control group. 

## 4. Discussion

The present study’s aim was to test the positive impact of the CAPEDP perinatal home-visiting program on maternal disruptive communication when the child reached the age of 12 months. In general, our findings underline that high poverty levels, premature delivery, or the accumulation of more than five risk factors are associated with higher rates of maternal disruptive communication (MDC) in our sample. They also show that women in the intervention group were 69% less likely than those in the usual care group to present MDC when interacting with their infants, and this when low income and prematurity variables were taken into account in the regression analysis.

Overall, the present results underline the importance of implementing intervention programs that specifically aim to decrease caregivers’ disruptive communication, so that the risk of disorganized attachment may consequently be diminished. According to Lyons-Ruth et al. [8], attachment disorganization emerges from the absence of fear arousal regulation in the infant, typically in the case of very insensitive caregivers. Additionally, when multi-risk parents face difficulties, such as financial problems or losing their job or their partner, the attachment relationship may be directly affected, the basic helplessness and insecurity of the caregiver having a significant impact on the child [31]. 

### 4.1. Maternal Disruptive Communication and Risk Factors

Cyr et al. [7] discussed the role of *independent* versus *cumulative* effects of socioeconomic factors on attachment disorganization in infants, this question has never been examined with regard to parental disruptive communication. From the *independent effects* point of view, the present study observes, as mentioned above, that enduring poverty and premature delivery are associated with maternal disruptive communication. As expected, the intrinsic stress connected with prematurity seems to directly affect parental behavior, giving place to more disrupted communication. The present results also underline the particular importance of seeing socioeconomic vulnerability as a risk factor for maternal disruptive communication, with poverty directly influencing parental behavior quality. This is in line with other studies, which have shown that low income may intensify the daily stress levels of parents and, consequently, not only decrease sensitive parenting behavior [32], but also increase disruptive communication and negatively affect the child’s attachment quality. It should be emphasized that it is the disruptive parenting, rather than poverty itself, that seems to be the key factor affecting outcomes for children.

Considering the cumulative hypothesis in the present study, disruptive mothers present a higher number of independent risk factors and are more likely to accumulate more than five risk factors. Several authors have examined cumulative risk as a predictor for the development of attachment security and disorganization [7,14,16,17]. However, cumulative risk as a predictor of parental disruptive communication has never, to our knowledge, been explored before. In the present study, stress-inducing factors, such as poverty and premature birth, are clearly associated with maternal disruptive communication. However, cumulative risks may well have a significant exacerbating influence. Even if independent risk factors seem to be sufficient in themselves to increase the disruptive communication of caregivers, the accumulation of any type of maternal risk factors (socioeconomic, psychopathologic, infant-related, parenting-related) increases the frequency of maternal disruptive communication. The importance of these results should not be underestimated, for they encourage us to devise better targeted prevention programs, which, in turn, will help promote infant mental health and attachment quality in vulnerable populations. 

### 4.2. Impact of the Intervention Program on Maternal Disruptive Communication

The present results confirm that the CAPEDP home-based program has helped to decrease maternal disruptive communication in a vulnerable French population. The intervention mothers have 59% lower probability of having disruptive communication than care-as-usual mothers. When adjusting for “low income” and “giving birth prematurely”, the probability of not having disruptive communication increases to 69%, allowing us to conclude that the presence of these kinds of risk factors might have diminished the program’s effects. 

The results of the present program are clearly related to its two-fold aim: a) promoting maternal sensitivity and diminishing disruptive maternal communication, and b) helping mothers to better handle contextual issues, such as parenting stress, social support, home safety, and job placement. Out in the community, intervention psychologists often realized that sometimes the better strategy for protecting the child was to prioritize socioeconomic issues. Only after the caregiver’s contextual or psychological stress was addressed, could a fruitful focused effort to enhance parenting skills be possible. This temporary adjustment was, however, of a strategic nature since the program’s focus on parental–infant interaction quality was never ignored. Focusing on intrusive, role-reversal, or withdrawal maternal behavior was always a concern, being one of the main initial goals of the intervention. Even so, the whole intervention could have been at risk if the multiple sources of caregiver stress had not been addressed and dealt with.

From a clinical point of view, the present findings highlight the importance of interventions specifically aiming to reduce the caregiver’s disrupted communication in populations suffering from high contextual adversity. As we have seen, the accumulation of social and economic stressors, which is a reality that parents face in these environments, may increase parental stress, attenuating their ability to assist their child’s need for regulation of fearful arousal and distress, and, consequently, affecting attachment relationships [33,34]. In a clinical setting, mental health professionals should help parents to decrease maternal disruptive communication by improving their ability: (a) to detect and become more aware of their own disruptive communication, (b) to better regulate their own stress and, accordingly, (c) reduce their own attachment system activation in stressful interactions with the child, which leads to (d) more efficiently activating their own caregiving system when children manifest distress, fearful behavior, or disorganized behavior [33]. According to Madigan et al. [34], a decrease in disrupted maternal communication in the context of an intervention can as early as the second treatment session over the course of a five-session video-feedback intervention, tending to remain and to become more sustained as the treatment sessions progress. The clinical importance of this kind of program is related to the link mentioned above between maternal disruptive communication and attachment quality. Attachment quality being a significant determinant of infant mental health, the importance of early interventions targeting this variable is nowadays undeniable [1].

The positive results of the present study may also well be related to having recruited psychologists who were specifically trained in attachment issues and then carefully supervised throughout the program (see [35]). Although socio-contextual support from general mental-health workers is clearly of value, handling parental disruptive communication requires sophisticated technical skills that are specific to psychology experts. Knowing how to address and help process trauma, or the use of the video-feedback approach [35] are good examples that illustrate our point. Using these approaches, disruptive parents were able to better recognize their own disruptive communication toward their children, as well as their subsequent sensitive and positive behavior, facilitating the reparation of these disruptive periods of interaction with the child. In the literature, authors such as Clarke, King, and Prost [36] claim that psychological interventions provided by non-specialists are beneficial for perinatal mental disorder programs, because they can broaden the range of access to these interventions. However, Moss et al. [20] highlight the fact that extensive training and supervision of intervention professionals are essential components for the success of these kinds of programs. Clarke et al.’s [36] perspective is interesting when programs are general and, hence, less technically demanding. Still, when it comes to correcting highly dysfunctional caregiver–infant interactions (such as parental disruptive communication and infants’ disorganized attachment), it is our opinion that highly trained and supervised psychologists are essential components. 

The CAPEDP study’s ability to scale up its protocol should also be addressed. Heckman claimed that the “highest rate of return in early childhood development comes from investing as early as possible, from birth through age five, in disadvantaged families” [36] (p. 1). With regard to the present study, the global cost included EUR 990,000 for the implementation of the CAPEDP program and EUR 90,000 for the CAPEDP-A assessment, representing a EUR 2455 average investment per family. Clearly, prevention programs targeting high-risk populations have the potential to make a sizeable impact on parent and child well-being, as well as to substantially contribute to the productivity and prosperity of society at a relatively low cost. Initial investment costs are easily recovered. This recovery increases into adulthood, thanks to cost-savings by, for example, decreasing emergency room visits or less need for child protection, special education, and social services [37].

### 4.3. Study Limitations and Future Directions

The study presents several limitations. Firstly, the control group cannot be considered to have received “zero” intervention, since “usual care” in the French health and social care system is quite generous, especially when compared to many other countries. In future studies, the option for a control group drawn from the general population would probably be more accurate in identifying the intervention effects of the program. Secondly, women who were not fluent French speakers and, therefore, not capable of giving their informed consent to participate, or who were already receiving other types of clinical interventions, were not included in the study. Future studies should take into account the fact migrant populations are often highly represented in socially at-risk populations. 

Methodologically speaking, it has been suggested that coding disruptive parental communication during the Strange Situation procedure raises the possibility of common-method variance, leading to a risk of contamination. The likelihood of overlap, in the same video, between data that were expected to be found and the actual observation of the infant’s attachment behavior may increase. In the present study, the risk of contamination was controlled by using AMBIANCE coders who were not trained with regard to infant attachment behavior. Finally, when mother–infant dyads were assessed for maternal disruptive communication, only two-thirds of the total number of planned home visits in the home-visiting program had taken place. 

## 5. Conclusions

Although children from multi-risk families seem to be highly exposed to attachment disorganization, the association between socioeconomic vulnerability and maternal disruptive communication has received little attention from researchers. 

The present study sought to throw some light onto this question by examining the impact of the CAPEDP program with regard to reducing MDC. Some of the strengths of this intervention are related to the fact that: (a) interveners received a thorough grounding in attachment theory and developmental psychopathology; (b) they frequently addressed and facilitated the reduction in maternal trauma-related symptomatology; (c) they required extensive observational training to address accurately the different disrupted maternal communication manifestations. Our study also clearly supports the Moss et al.'s [20] idea that extensive training and frequent supervision are essential program’s components for intervention success.

Several policy implications of the current intervention merit consideration. Firstly, the study results support the importance of implementing intervention programs that specifically aim to decrease MDC, so that the risk of disorganized attachment may consequently be diminished. The fact that even though the control group did receive a significant level of care, with the home-visiting assessment process and the availability of the research team to help with any trouble that might be detected with a child or with a mother, the difference with regard to disorganizing behavior still remained significant. It is clearly worth reaching out to more vulnerable families, using targeted home-based preventive strategies supported by video interaction guidance. Secondly, even if it is clear that early interactions with caregivers shape the quality of parent–child attachment relationships, attachment stability in early childhood remains at risk, especially if the quality of the parent–child relationship is compromised due to changes in family circumstances or the caregiving environment [1]. Thus, although the program had a significant effect with regard to reducing MDC, this positive outcome may not be enduring if continued intervention services for these families are not provided. Follow-up sessions should occur after the regular completion of a protocol in order to increase the probability that treatment results are kept over time. In our case, we have invested in a second phase of the project (CAPEDP-A phase II), up to the child’s fourth birthday, where we’ll be able to evaluate our families’ benefits of receiving continued intervention during the early childhood period. Finally, the study demonstrates that vulnerable first-time families do respond positively to an offer of support and guidance for their child, whatever their socioeconomic level and ethnic group. As Fraiberg [38] puts it, parents will do much more for their baby and its welfare than they would do for themselves.

Since parental disruptive communication correlates with children’s attachment disorganization, the present results confirm that the use of a two-fold approach in intervention programs, addressing both parental disruptive communication and the risk-factor accumulation perspective is of extreme relevance.

## Figures and Tables

**Table 1:** Risk factors at inclusion of mothers who declined to participate compared with those of women who accepted to participate in CAPEDP-A sub-study.

		Declined CAPEDP-A (*n* = 248) *n* (%)	Accepted CAPEDP-A (*n* = 119) *n* (%)	*p*-Value
**Group**	Care as usual	131 (52.8)	52 (43.7)	
	intervention	117 (47.2)	67 (56.3)	*p* = 0.10
Age	Min/Max	16.0/26.0	16.0/26.0	
	Mean (std)	22.3 (2.4)	22.3 (2.5)	*p* = 0.85
Access to free health care	Yes	120 (49.0)	50 (42.4)	
	No	125 (51.0)	68 (57.6)	*p* = 0.24
<9 years of education	Yes	43 (17.5)	18 (15.1)	
	No	203 (82.5)	101 (84.9)	*p* = 0.57
Marital status	In couple	132 (53.9)	71 (59.7)	
	Single	113 (46.1)	48 (40.3)	*p* = 0.30
Monthly income < EUR 840	Yes	105 (44.9)	45 (39.5)	
	No	129 (55.1)	69 (60.5)	*p* = 0.34
Nationality	French	141 (57.3)	70 (58.8)	
	Other	105 (42.7)	49 (41.2)	*p* = 0.79
Planned pregnancy	Yes	145 (59.2)	81 (68.1)	
	No	100 (40.8)	38 (31.9)	*p* = 0.10
Tobacco and drug use	Yes	61 (24.9)	33 (27.7)	
during pregnancy	No	184 (75.1)	86 (72.3)	*p* = 0.56
Depressive symptomatology	Mean (std)	10.8 (5.7)	10.6 (5.5)	*p* = 0.70

**Note:** For certain variables in the above table, the total number of responses is less than the total number of subjects due to missing data.

**Table 2:** Sample demographics and risk factors at inclusion across the intervention and control groups.

	Intervention Group (n = 63) n (%)	Control Group (n = 51) n (%)
**Child age** mean, SD in months	14.3 (2.9)	14.0 (2.7)
**Maternal age** mean, SD in years	22.1 (2.5)	22.5 (2.5)
<20 years old	20 (37.7) **	14 (20.9)
**Infant gender** males	21 (46.7)	27 (45.8)
**Marital status** married/living together	34 (65.4)	37 (55.2)
**Educational level** <= 9 years	9 (17.3)	9 (13.4)
**Monthly income** < EUR 840	20 (40.8)	25 (38.5)
**Tobacco/drug use during pregnancy**	24 (35.8) *	9 (17.3)

**Note:** There were no significant differences in sociodemographic variables between the intervention and care-as-usual groups, except for mothers younger than 20 y-o (** *p* = 0.01) and tobacco/drug use during pregnancy (* *p* = 0.03).

**Table 3:** Risk factors and maternal disruptive communication (MDC) at inclusion.

Risk Factors	MDC (*n* = 48) *n* (%)	No MDC (*n* = 66) *n* (%)	*p*-Value
***Socioeconomic Risk***			
At least one	37 (77.1)	47 (71.2)	*p* = 0.40
Total number (mean; SD)	1.5 (1.1)	1.2 (1.0)	*p* = 0.04 *
<9 years education	9 (19.1%)	8 (12.1%)	*p* = 0.32
Low income < EUR 834 month	26 (54.2%)	22 (33.8%)	*p* = 0.04 *
Single parent	11 (22.9%)	16 (24.2%)	*p* = 0.91
***Psychopathologic Risk***			
At least one	32 (66.7)	42 (63.6)	*p* = 0.55
Total number (mean; SD)	1.2 (1.0)	1.1 (1.0)	*p* = 0.31
EPDS > 11 à T1	23 (48.9)	23 (35.4)	*p* = 0.14
SCL > 0.80	25 (53.2)	27 (41.5)	*p* = 0.17
Tobacco/alcohol/substance use during pregnancy	10 (21.3)	21 (31.8)	*p* = 0.37
***Parenting Risk***			
At least one	43 (89.6)	53 (80.3)	*p* = 0.18
Total number (mean; SD)	1.9 (1.1)	1.5 (1.1)	*p* = 0.08
Previous pregnancy not brought to term	21 (44.7)	23 (34.8)	*p* = 0.27
Planned pregnancy	30 (63.8)	48 (72.7)	*p* = 0.33
Loss of key attachment figure < 11 years-old	16 (39.0)	27 (44.3)	*p* = 0.52
Less than 20 years old	31 (66.0)	50 (75.8)	*p* = 0.43
***Infant Risk***			
At least one	15 (31.3)	23 (34.8)	*p* = 0.94
Total number (mean; SD)	0.3 (0.5)	0.3 (0.5)	*p* = 0.73
Premature	6 (13.0)	2 (3.2)	*p* = 0.04 *
Tobacco/alcohol/substance use during pregnancy	10 (21.3)	21 (31.8)	*p* = 0.37
***Cumulative Risk Factors***			
Total number (mean; SD)	4.4 (2.1)	3.4(2.0)	*p* = 0.01 **
At least 3	40 (83.3)	49 (74.2)	*p* = 0.28
At least 4	33 (68.8)	35 (53.0)	*p* = 0.07
At least 5	24 (50.0)	20 (30.3)	*p* = 0.02 *
Number of home visits (mean; SD)	18.5 (7.05)	19.1 (7.7)	*p* = 0.75

**Note:** In order to ensure the independence of these risk variables, correlation analyses were conducted between them. Only those that were not significantly correlated were included in the analysis. (** *p* < 0.01 and * *p* < 0.05).

**Table 4:** Maternal disruptive communication by group.

	Intervention Group n = 63	Control Group n = 51	Total N = 114	χ^2^ (dl)	*p*-Value
**Disrupted**	20 (31.7%)	27 (51.9%)	47 (41.2%)	4.45 (1)	0.04 *
**Not disrupted**	43 (68.3%)	24 (48.1%)	67 (58.8%)		

(* *p* < 0.05).

**Table 5:** Impact of the CAPEDP intervention on maternal disruptive communication, taking into account the significant risk factors for MDC.

Variables	OR	95% CI	*p*-Value	Effect Size
Impact of CAPEDP intervention on MDC	0.44	0.28–0.95	0.04	**2.27**
Impact adjusting for significant risk factors (low income, premature baby)	0.31	0.10–0.93	0.04	**3.23**

## Data Availability

Data supporting reported results can be provided by the authors.
